# Tuning the Localized Microenvironment near a Continuous Glucose Meter to Ensure Monitoring Accuracy and Longevity by Plasma-Induced Grafting Zwitterionic Brushes

**DOI:** 10.1021/acssensors.4c01921

**Published:** 2024-12-05

**Authors:** Syuan-Jia Shin, Pei-Chen Lo, Yen-Ting Wu, Huai-Hsaun Shao, Dai-Jin Li, Yung-Cheng Weng, You-Yin Chen, Ta-Chung Liu

**Affiliations:** † Department of Biomedical Engineering, National Yang Ming Chiao Tung University, 155 Lin-Ong St., Taipei, Taiwan 11221, ROC

**Keywords:** continuous glucose monitoring, zwitterionic brush, atmospheric plasma-induced grafting, microenvironmental pH, antifouling

## Abstract

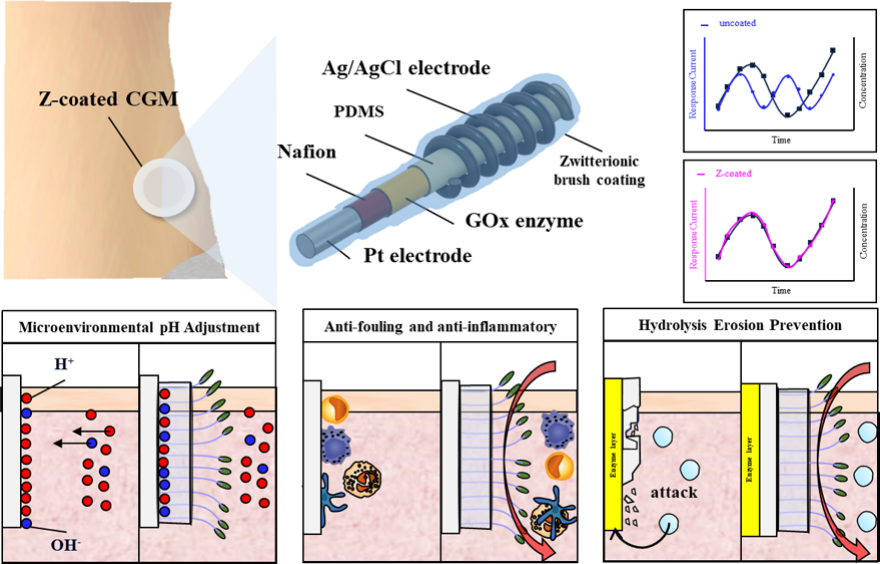

Diabetes mellitus is a metabolic disorder that affects millions of individuals worldwide. Continuous glucose monitoring (CGM) offers a prevalent method for continuously monitoring interstitial glucose levels instead of traditional self-monitoring of blood glucose (BG), eliminating the need for finger pricking and providing only discrete data. However, challenges in accuracy persist in CGM, including substantial noise interference and tissue fluid erosion, as well as the pH fluctuations in the localized ISF microenvironment during acute inflammation periods. Herein, we reported a facile atmospheric plasma-induced grafting technique to surface functionalize a zwitterionic brush coating on the sensor, with the aim to adjust the sensor's microenvironmental chemistry. The zwitterionic brush-coated CGM (Z-coated CGM) could regulate pH values with a good glucose response in the pH range from 6.2 to 7.6 and a prolonged sensor life over the uncoated sensor. We evaluated the rat practice that the Z-coated CGM consistently outperformed the uncoated in tracking BG fluctuations, with higher correlation coefficients and significant noise reduction for both non-recalibration and recalibration. This technology holds substantial implications for subcutaneous embedded glucose monitors and facilitates CGMs in achieving independence from routine BG fingerstick calibrations.

Diabetes, a metabolic disease in which blood glucose (BG) regulation is hindered, affects hundreds of millions of people worldwide, many of whom remain undiagnosed.^1−3^ The current standard for clinical treatment of patients with diabetes is self-monitoring of BG, measuring BG through fingertip blood collection multiple times a day and then injecting insulin as needed to adjust blood sugar in the normal range.^4−6^ However, self-monitoring of BG can only access discrete BG data. It cannot reflect BG fluctuations over time, coupled with the pain associated with repeated finger prick testing, making self-monitoring BG a disadvantageous practice for patients and doctors.^7,8^

Continuous glucose monitoring (CGM) is a wearable medical device that can monitor glucose over a decent period.^9−11^ By implanting a patch coupled with a soft needle as the glucose sensor under the skin, the glucose level in the subcutaneous tissue interstitial fluid (ISF) can be visited every 5–15 min. Therefore, compared with traditional extracorporeal BG meters, CGM provides continuous glucose measurements for real-time monitoring and significantly reduces physical pain for patients. However, enormous challenges remain in current CGM measurements, including (1) acute inflammation upon implantation, which produces significant noise during the initial 72 h, while the specific mechanism remains unclear;^12,13^ (2) fibrotic capsule formation surrounding the sensor, caused by the wound healing process, which limits the transportation of analytes in the long-term implantation;^14,15^ (3) durability and safety of the adhesive;^16^ and (4) physiological environmental changes that may jeopardize the specificity of the enzyme, resulting in inaccuracy monitoring.^17,18^ Notably, the anomeric properties of glucose molecules, which are sensitive to environmental factors, can hugely impact detection accuracy, which is often overlooked. The ratio of α-D to β-D glucose anomers depends on temperature and pH value, as well as the concentration and solute, among which β-D glucose is preferred under neutral and alkaline conditions and α-D glucose is preferred under acidic conditions.^19,20^ This indicates that the detection of ISF glucose poses a severe challenge since the pH of ISF fluctuates from 6.6 to 7.6,^21^ not to mention the inflammatory nature of the initial implantation that can be as low as 6.2,^22,23^ yet blood has a constant pH (7.35–7.45). This means that glucose detection using an anomeric-selective enzyme (such as glucose oxidase enzyme GOx, which is specific only for β-D glucose) may not produce reliable measurements.^24^ Therefore, the pH of the sample and the localized implanted environment must be known to obtain accurate glucose readings technically by postcalibrations.^25,26^

To overcome this challenge, we developed atmospheric plasma-induced grafting of a zwitterionic brush coating on the homemade CGM surface to regulate the microenvironment around the sensor, mainly aiming to buffer and regulate the localized pH values. This stabilizes the configurations of β-D glucose near the electrode and remains accurate during the acute inflammatory period. The purpose of using atmospheric plasma to induce grafting of a zwitterionic brush coating is not only to minimize the boundary barrier between the glucose confinement layer and the zwitterionic brush coating layer so that the diffusion rate is balanced to maintain the integrity of the glucose confinement^27^ but also to decrease interfacial resistance compared to other direct physical assembly strategies, such as layer-by-layer coating.^28,29^ Besides, atmospheric plasma-induced grafting is a mature technology that can be easily integrated into industrial production.^30,31^ Furthermore, the zwitterionic coating has been reported to show services in many implantable devices due to its ultralow fouling properties, attributed to the creation of a highly hydrated environment and the establishment of a steric exclusion barrier to block the attachment of proteins.^32−35^

Compared to the uncoated CGM, the zwitterionic brush-coated CGM (Z-coated) showed the adjusting ability of microenvironmental pH with good linear responses in the pH range of 6.2–7.6 and survived for 12 days at 37 °C in protein-rich serum. In the rat experiment, the Z-coated CGM exhibited a better follow-up (with higher correlation coefficients) and significantly reduced noises to BG measurements over the uncoated. This technology and the used zwitterionic material are of great significance for subcutaneous embedded glucose monitors and potentially help CGMs gain independence from the requirement of simultaneous BG testing for calibration since it effectively provides the measurement accuracy of subcutaneous glucose level, especially in the acute inflammatory phase, which is crucial for standalone monitoring devices for diabetic patients.

## Experimental Section

### Fabrication of CGM Sensor

For the uncoated CGM, the polytetrafluoroethylene (PFA)-coated Pt wire (0.2 mm o.d.; 0.12 i.d., A-M Systems), where the PFA layer was peeled off at the front end for 2 mm, acted as the working electrode. A silver wire (0.1 mm o.d., Ing-Jing Precise Industrial Corp.) was tightly wrapped around the PFA layer, covering about 3 mm of its length. The silver chloride layer (AgCl) was formed by passing current (0.4 mA/cm^2^) for 1 h through the silver wire in a stirred 0.1 M HCl solution (J.T. Baker) and then was rinsed with deionized water for 6 h. The Ag/AgCl wire worked as the reference/counter electrode. The exposed working Pt wire region was dipped in 5% (w/w) Nafion solution (Morr Technology Co., Ltd.). A DC of 3 V was applied to the working electrode for 10 s to form a thin Nafion layer, which was then dried in air for 2 h. The GOx (243 units/mg, Amano Enzyme Inc.) was physically adsorbed by soaking the working electrode region in GOx solution (80 mg/mL) and allowed it to dry at room temperature for 10 min. To immobilize the enzyme, the electrode was exposed to glutaraldehyde vapor generated from 25% glutaraldehyde solution (Alfa Aesar) placed at the bottom of an enclosed glass chamber for 12 h at 37 °C. The electrode was then rinsed in deionized water and dried in air for 2 h. The electrode was then dipped in the PDMS solution (Uniregion Bio-Tech) and placed in an oven at 50 °C to cross-link. The PDMS layer served as the glucose confinement layer and the protective biolayer.

### Protocol of Plasma-Induced Grafted Zwitterionic Brush Coating of CGM

For the Z-coated CGM, the CGM was pretreated by an atmospheric plasma (8 W, 15 s) to clean and generate free radicals on the surface. Then, the electrode was immersed in 10 wt % [2-(methacryloyloxy)ethyl] dimethyl-(3-sulfopropyl) ammonium hydroxide (SBMA; Sigma-Aldrich) monomer solution for 2 h. The CGM was further treated with an atmospheric plasma to initiate grafting polymerization. Finally, the as-prepared CGM was washed to remove the residual SBMA monomers and ungrafted SBMA polymers.

### Characterizations of CGMs

All electrochemical measurements were implemented using an 8-channel potentiostat workstation (OctoStat30, Ivium, Netherlands). The surface morphology and composition of CGMs were carried out using field-emission scanning electron microscopy (JEOL JSM-7600F), equipped with an energy-dispersive X-ray spectroscopy (EDX) analyzer. X-ray photoelectron spectroscopy (XPS; ULVAC-PHI (Quantes), Japan) with an Al–Kα X-ray source was used to analyze the binding chemistry between the PDMS layer and the zwitterionic brush coating.

### In Vitro Glucose Permeability

In vitro glucose permeability data were obtained using an H-shaped diffusion cell apparatus maintained at 37 °C. The membranes were immersed in phosphate-buffered saline (PBS, pH 7.4) for 3 h to attain equilibrium and sandwiched between the donor and receiver chambers of the H-cell. Under 5 mL of glucose solution with a concentration of 30 mM in the donor chamber and 5 mL of PBS in the receiver chamber, the concentration changes were measured in the two chambers every hour with homemade glucose sensors. The diffusion coefficient of glucose through the membrane was calculated by using eqs 1 and 2:
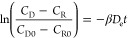
1
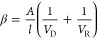
2where β is the cell constant; *D*_e_ is the effective diffusion coefficient; *C*_Do_ and *C*_Ro_ are the initial glucose concentrations in the donor and receiver chambers, respectively; *C*_D_ and *C*_R_ are the glucose concentrations at time *t* in the donor and receiver cells, respectively; *A* is the area of diffusion; *l* is the membrane thickness; and *V*_D_ and *V*_R_ are the volumes of donor and receiver cells, respectively.

### In Vitro Glucose Measurements

Unless otherwise stated, the uncoated and Z-coated CGMs were conducted using a two-electrode setup with the exact analysis/measurements, and the liquid sample amount in a voltammetry cell for all sensors was 2 mL. For measuring in vitro glucose levels, CGMs were evaluated by stepwise chronoamperometry (CA) at 0.6 V (vs Ag/AgCl) of glucose concentration from glucose-free to 30 mM. For the phantom commercial testing, CA was conducted by obtaining 10 s glucose signals at 0.6 V (vs Ag/AgCl) and rested for 140 s at open-circuit potentials (OCPs) in glucose-containing PBS at room temperature. Note that the glucose concentration was increased by 5 mM after every intermittent step (150 s). For the interference study, tests were conducted by sequential addition of 5 mM glucose, 0.1 mM acetaminophen (AP, Sigma-Aldrich), 0.11 mM ascorbic acid (AA, Sigma-Aldrich), 0.4 mM uric acid (UA, Sigma-Aldrich), and 5 mM glucose. CGMs were implanted in agarose hydrogels (Sigma-Aldrich) containing different glucose concentrations from glucose-free to 30 mM for respective CA tests to mimic sensor implantation in tissue. The long-term stability of CGMs was measured in glucose-containing protein-rich porcine serum from glucose-free to 30 mM each day for 12 consecutive days at 37 °C.

### Investigation of Microenvironmental pH of CGM

For evaluating the tuning ability of the localized microenvironment pH near the Z-coated CGM, OCPs were recorded for both the uncoated and Z-coated CGMs incubated in PBS with various pH values from 5.8 to 7.4 to present the relationship between the OCP and pH. The in vitro glucose measurements were also tested by CA of glucose concentration from 2 to 20 mM at pH values of 6.2 and 7.6, respectively.

### In Vivo ISF Glucose Monitoring in Animals

Eight-week-old male Sprague–Dawley rats (BioLASCO, Taiwan) were used and housed in a climate-controlled environment with 12 h light and 12 h dark cycles (weighing approximately 250 g during the experiment). Individuals were randomly selected to receive STZ (streptozotocin)-induced diabetes. Before administration of STZ, rats underwent a 12 h fasting period and were kept hydrated to prevent dehydration. A freshly prepared STZ solution at a 50 mg/kg dose was then administered via tail vein injection. BG levels should be monitored regularly for 48 h after injection. A BG concentration exceeding 16.7 mmol/L indicates the successful induction of diabetes.

CGM approved all testing procedures. Rats were anesthetized using a 1:1 mixture of depositor (Zoetis) and telazol (Zoetis) and fixed on an operating plate (Figure S1). Then, the uncoated and Z-coated CGMs were implanted in the dorsal region. Before animal experiments, sensors were soaked in PBS for 1 h before implantation. For healthy SD rats, after anesthesia, an incision was made in the inguinal skin to expose the femoral vein, where a syringe hose was inserted. At the same time, uncoated and Z-coated CGMs were implanted 1 cm lateral to the spinal cord and between the shoulder blades. After 30 min, 0.4 mL of a 4 M glucose solution was injected to induce BG changes. For diabetic SD rats, the uncoated and Z-coated CGMs were also implanted 1 cm lateral to the spinal cord and between the shoulder blades after anesthesia. In contrast to the healthy rats, diabetic rats were injected with five units of insulin into the left hind leg using an islet syringe (FlexPen NovoRapid, Novo Nordisk A/S, Denmark) 30 min after the start of the experiment to stimulate BG changes. The ISF glucose concentration was evaluated using CGMs by CA (140 s for rest at the OCP and 10 s for recording) over 90 min. BG was measured every 10 min using BG kits (GlucoLeader GV01, HMD Biomedical, Taiwan). The blood was collected from the tail vein by using a lancet.

### Data Analysis and Statistics

The recalibration method mainly referred to Kai et al. and published reports.^44−46^ For the technique of delayed compensation of processing in vivo glucose monitoring, CA measurements recorded the response current every 150 s. In comparison, the venous BG was measured every 600 s using BG kits. Based on the literature and clinical reports, there is a 5–15 min (300–750 s) delay between BG and ISF glucose levels. Therefore, the ISF current data points were shifted forward by 300, 450, 600, and 750 s, respectively. Excel software was used to calculate the correlation coefficient between the ISF current data and BG levels simultaneously. A linear regression relationship was calculated from the correlation profiles to convert the ISF current data to the corrected glucose levels. The deviation (difference) between the corrected glucose levels and BG was calculated using the following formula:
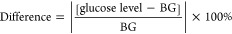
where difference represents the percentage difference between the glucose levels transformed from the sensor current with and without using recalibrated linear regression formulas.^36^

### Histology Analysis

The tissue surrounding the implanted sensor was cut and sliced with a thickness of 5 μm and immersed in formalin. The H&E and Masson trichrome staining was conducted by the Zoetis Animal Pathology Testing Center (Taiwan) and observed at different magnifications using a PANNORAMIC Flash DESK DX (3DHISTECH, Hungary).

## Results and Discussion

In this work, the PDMS layer, serving as the glucose confinement layer and oxygen permeable layer on the homemade CGM, is functionalized with a zwitterionic brush coating via atmospheric plasma-induced grafting. The aim is to reduce monitoring noises during the acute inflammatory period by establishing the ability to adjust the sensing microenvironment to eliminate the anomeric effect of glucose and prolong the longevity of the sensor.

**Figure 1:**
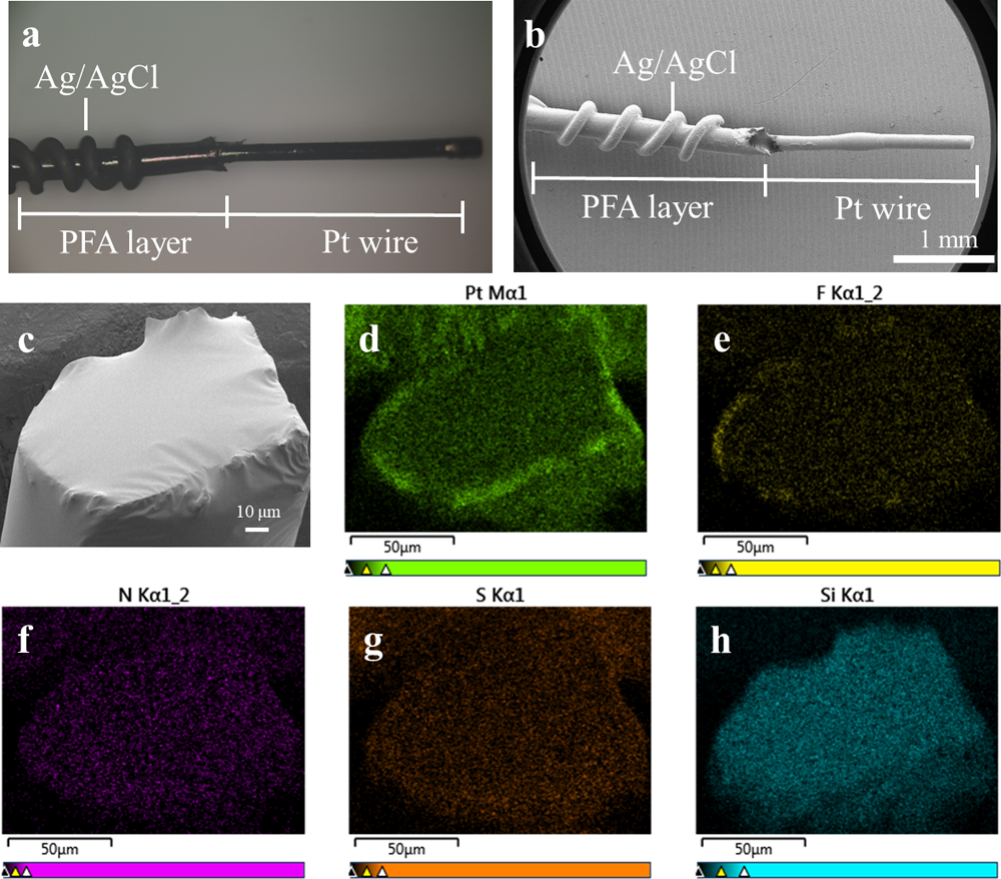
(a) Optical image and (b) SEM image of the Z-coated CGM. (c–h) SEM/EDX mapping was used to conduct a qualitative analysis of the basic parameters for each component.

The homemade microinvasive CGM, modified from Wilson’s design,^37,38^ consists of a conductive Pt wire electrode coated by a thin PFA insulative layer embedded with the Nafion layer that serves as the anti-interference layer and the glucose-specific enzyme glucose oxidase (GOx). The PDMS outer layer allows small molecules, such as glucose, O_2_, and H_2_O_2_ to diffuse through the layer while immobilizing GOx. The GOx catalyzes glucose oxidation to gluconolactone and produces H_2_O_2_, which then changes the electrical current of the electrode. The Ag/AgCl, reference/counter electrode, was wired to the end of the sensor (Figure 1a,b). Each component of the CGM was qualitatively analyzed through SEM/EDX. The element diagram showed that the representative elements of the sensor were platinum (Pt), fluorine (F), nitrogen (N), sulfur (S), and silicon (Si), indicating that the CGM was successfully assembled with the respective coatings (Figure 1c–h).

**Figure 2:**
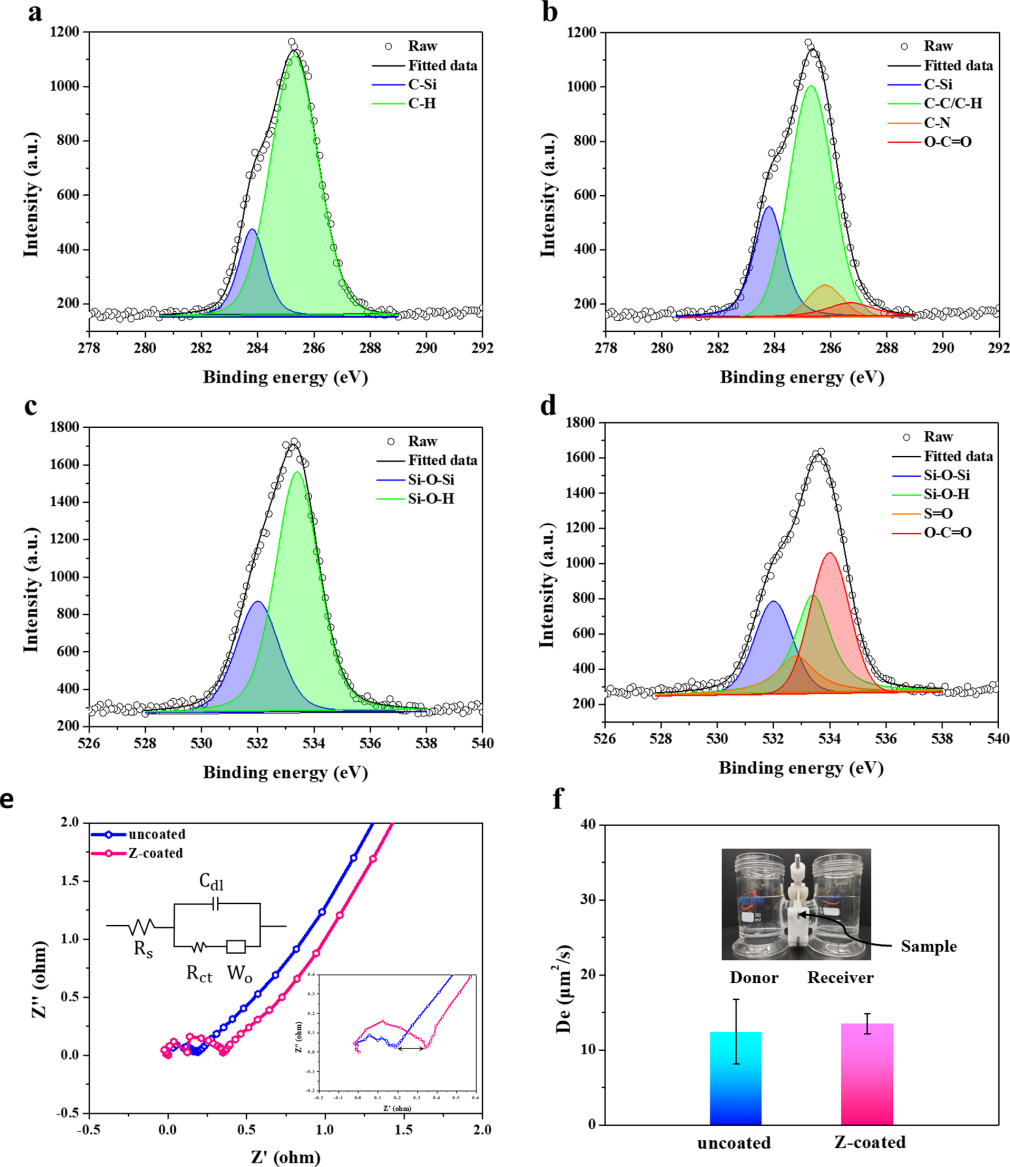
High-resolution XPS profiles show the bonding chemistry for (a) C 1s of uncoated, (b) C 1s of Z-coated CGM, (c) O 1s of uncoated, and (d) O 1s of Z-coated. (e) Nyquist plot of uncoated and Z-coated sensors (inset showed the equivalent circuit model). (f) Setup for a H-shaped diffusion cell for measuring glucose permeability with the corresponding diffusion coefficient (*D*_e_). Data are presented as mean ± s.d. (*n* = 3).

High-resolution XPS was implemented to examine the binding chemistry. The full XPS spectrum is shown in Figure S2. Compared to the uncoated CGM, the C 1s spectra of the Z-coated CGM showed a decrease in the binding energy of the PDMS C–H bonds, and new C–N and O–C=O bonds were found at 285.8 and 286.7 eV, indicating the presence of the pSBMA coating (Figure 2a,b). Additionally, in the O 1s spectra, a significant reduction in the Si–O–H bonds of PDMS was observed after plasma treatment, with new S=O and O–C=O bonds appearing at 532.8 and 534 eV, which are attributed to the pSBMA coating (Figure 2c,d). The decrease of C–H in C 1s and Si–O in the O 1s spectra revealed that the PDMS bonds on the sensor surface were disrupted, potentially forming bonding with the C=C of pSBMA at these sites, coupled with the existence of those signature bonding of SBMA, as evidence of the bonding between PDMS and the pSBMA coating.

Electrochemical impedance spectroscopy is a subtle characterization technique used to study the resistance/impedance of electrode interface behaviors. The charge-transfer resistance (*R*_ct_) generally reflects the resistance of the electrode surface to redox reactions or sometimes could be seen as a simple interfacial barrier on the electrode surface when no redox reaction occurs in the electrochemical system. In the low-frequency region (the end of the Nyquist plot), the straight line indicates diffusion behaviors defined as Warburg impedance (*W*_o_). It can be seen from the Nyquist plot (Figure 2e) that after grafting, there was a minimal increased resistance from 0.19 to 0.34 Ω, illustrating the existence of a thin pSBMA layer. Moreover, it can be found that the slopes of the Warburg line were almost the same between the uncoated and Z-coated CGMs, demonstrating that the efficiency of substance diffusion remains unaffected for the Z-coated sensor. To further corroborate that the surface functionalization of the zwitterionic coating does not affect the inherent diffusion ability of PDMS, diffusion coefficients (*D*_e_) of uncoated and Z-coated PDMS membranes were tested by using an H-shaped diffusion cell. The results indicated that the *D*_e_ of both samples showed limited changes, confirming that the surface functionalization does not impair the diffusion capacity of PDMS (Figure 2f).

**Figure 3:**
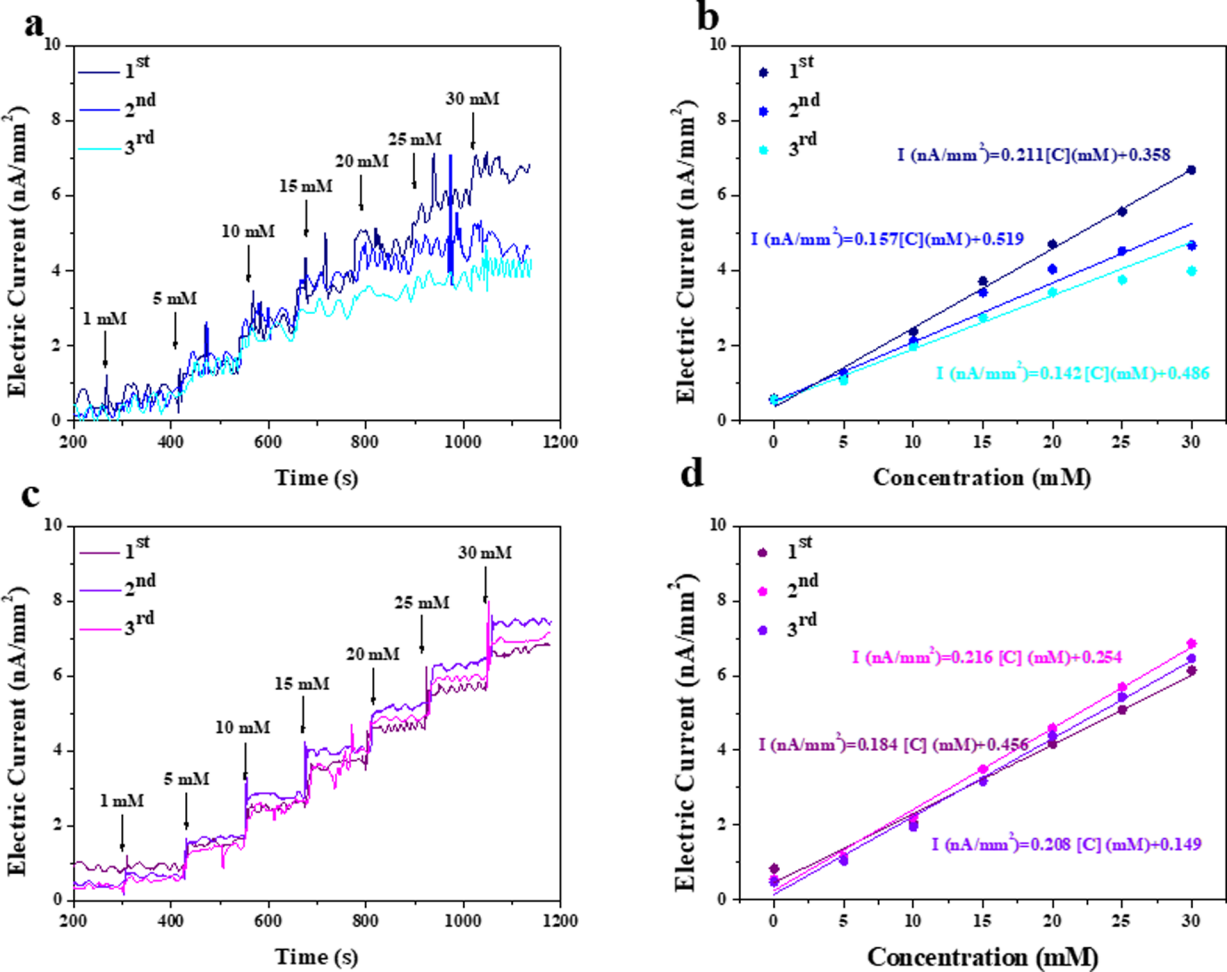
(a,b) Sensor sensitivities of the uncoated CGM and (c,d) of the Z-coated CGM performed by the CA profiles tested stepwise, followed by continuous glucose injection from glucose-free to 30 mM at 0.6 V (vs Ag/AgCl).

The in vitro responsiveness of uncoated and Z-coated CGMs to different glucose concentrations was recorded by gradually changing the glucose concentration from 0 to 30 mM at 0.6 V (vs Ag/AgCl) (Figure 3). The uncoated and Z-coated CGMs showed similar values of the limit of detection (LOD) with 0.13 and 0.15 mM, respectively, indicating that the zwitterionic coating does not impact the LOD, attributed to the evidence of the similar glucose diffusion behaviors for both uncoated and Z-coated. In three consecutive experiments, Z-coated CGMs showed a continuous increase in current with the constant addition of glucose, showing their stable response to glucose. However, the uncoated CGM exhibited significant noise, especially in higher-glucose-concentrated solutions, possibly due to the imbalance of diffusion at interfaces in such a tiny sensor. In contrast, the zwitterionic brush coating promotes a compact conformation of the hydration layer, ensuring stability and rapid glucose influx and/or efflux from the diffusive surface. In addition, when GOx catalyzed glucose, H_2_O_2_ was produced, and gluconic acid was produced as a byproduct. Through consecutive measurements, the accumulation of gluconic acid trapped in the PDMS matrix was not quickly diffused outward because of the poor transportation of substances between the hydrophobic PDMS layer and the aqueous glucose solution, leading to a potential localized anomeric effect of glucose and then impaired measurements.

**Figure 4:**
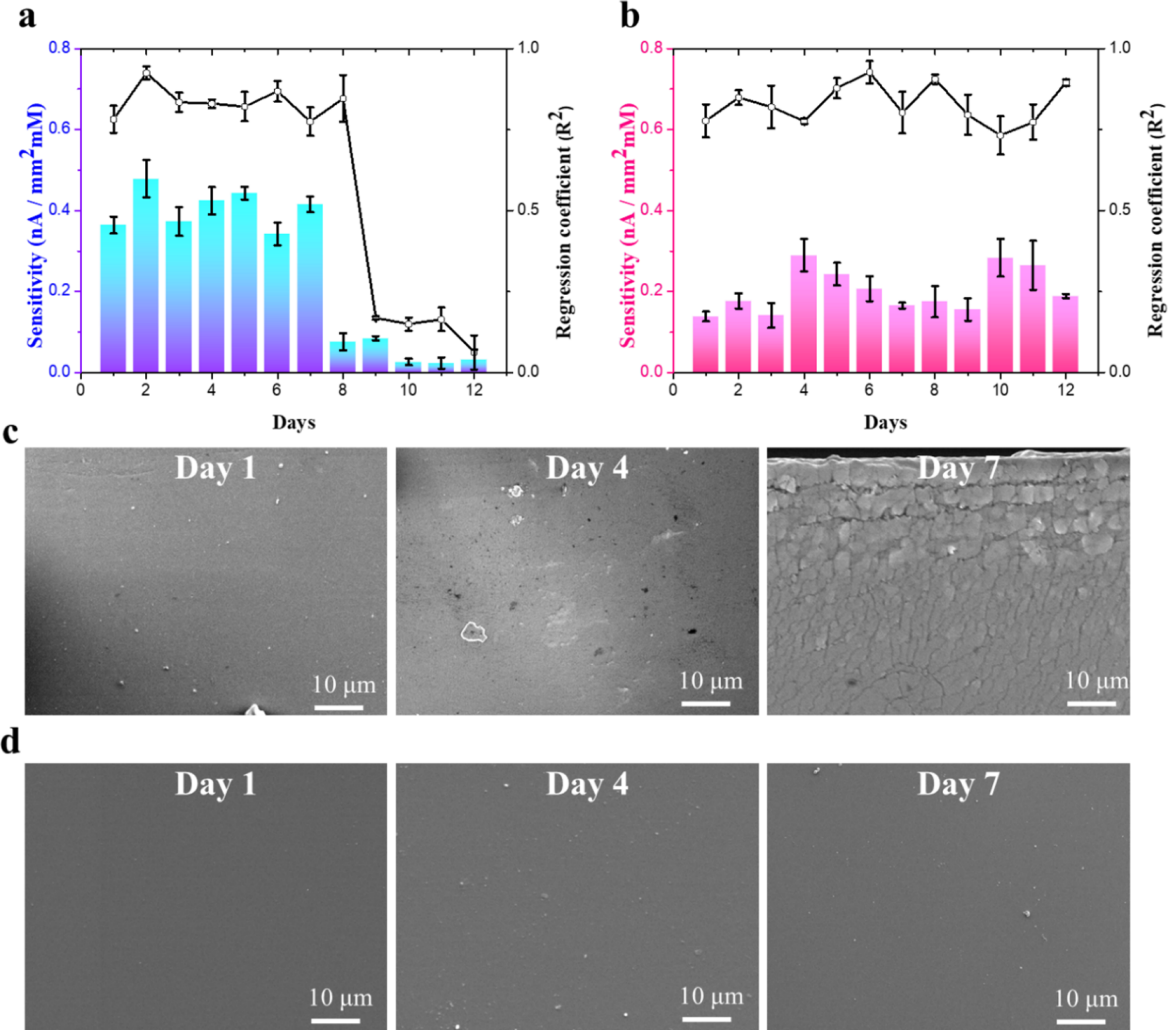
Investigating long-term sensor stability for (a) uncoated CGM and (b) Z-coated CGM depicted by sensor sensitivity and *R*^2^ versus time. Data are presented as mean ± s.d. (*n* = 3). SEM images of the (c) uncoated and (d) Z-coated phantom PDMS surface on the 1st, 4th, and 7th day in PBS at 37 °C.

To evaluate the long-term stability of the CGMs, the Z-coated CGM and the uncoated CGM groups (3 sensors were used in each group) were tested. They were kept in a physiologically protein-rich serum solution containing glucose at 37 ± 0.5 °C for 12 days. The sensitivity examination with glucose concentration ranging from glucose-free to 20 mM was conducted daily (Figure 4a,b). For uncoated CGMs, the sensitivity remained, and *R*^2^ (*R* squared, coefficient of determination) maintained above 0.75 until day 7. However, the Z-coated CGM lasted until the 12th day and maintained decent sensitivities. To verify the impact of the hydration layer formed by the zwitterionic brush coating on slowing the hydrolysis rate of PDMS, uncoated and Z-coated phantom PDMS samples were soaked in PBS at 37 °C for 7 days, and the surface morphology of the PDMS surface was observed by SEM. The results showed that uncoated PDMS exhibited erosion pits on the fourth day and even huge cracks on the seventh day. In contrast, the surface morphology of Z-coated PDMS on the seventh day showed no noticeable difference over time, indicating its ability to protect PDMS from bulk water hydrolysis (Figure 4c,d). It is attributed to the wealthy charged zwitterionic groups, providing intense hydration and antifouling functions for nonspecific protein adsorptions.^40^ The hydration layer buffers the violent erosion of external bulk waters and then extends the life of the enzyme layer,^41^ exhibiting a more reliable performance than the uncoated CGM group.

**Figure 5:**
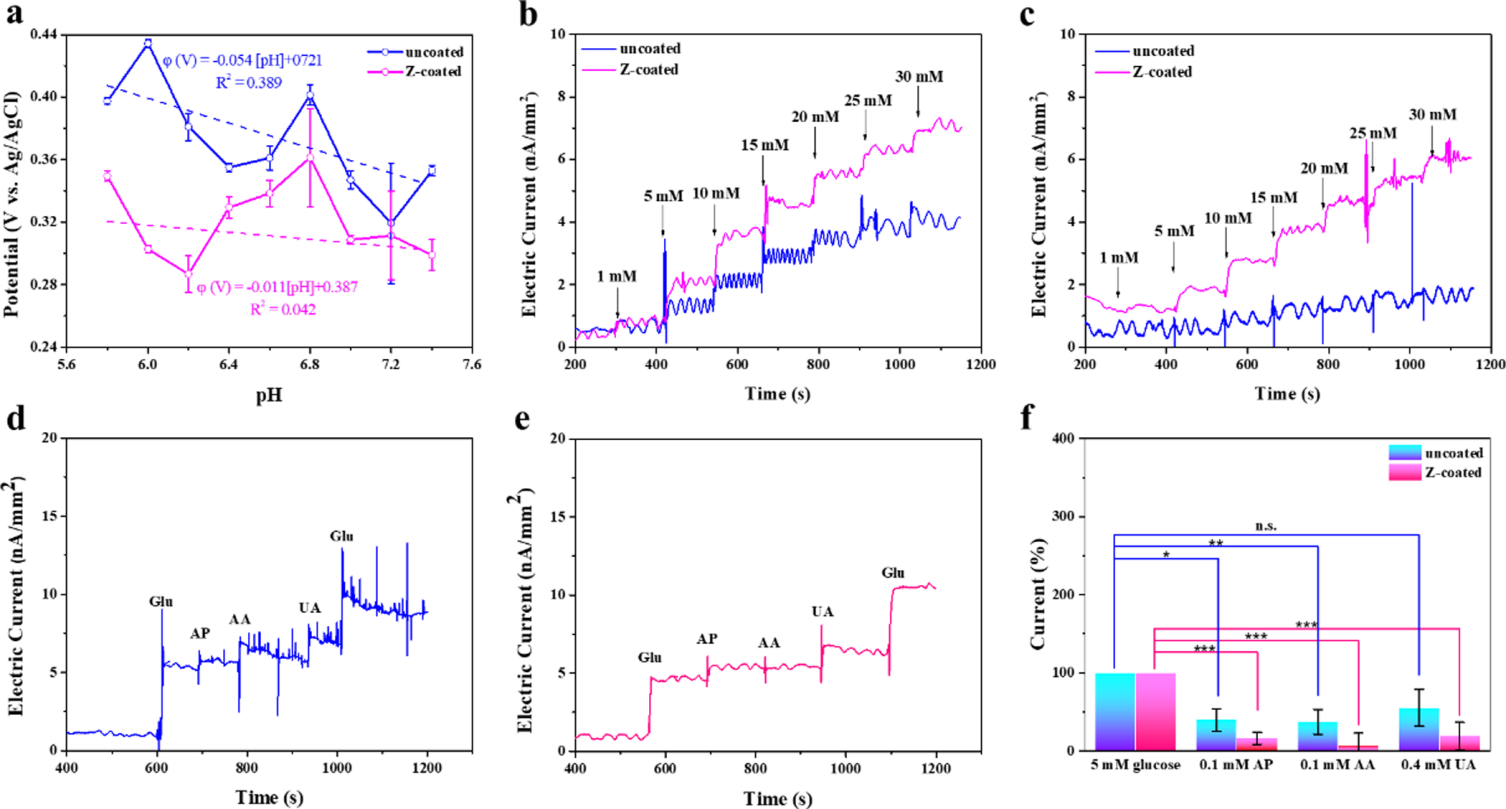
(a) Microneedle glucose sensor was incubated in 0.1 M PBS at pH 5.8–7.4 (pH intervals by 0.2) for 1 h of OCP measurement. (b,c) CA responses (at 0.6 V vs Ag/AgCl) for the uncoated and Z-coated CGMs by sequentially injecting from glucose-free to 20 mM at pH values of 7.6 and 6.2, respectively. In vitro interference study of (d) CA responses (at 0.6 V vs Ag/AgCl) for the uncoated and (e) Z-coated CGM by sequentially injecting 5 mM glucose, 0.1 mM AP, 0.11 mM AA, 0.4 mM UA, and 5 mM glucose. (f) Relative responsive current percentage for the uncoated and Z-coated CGMs with glucose and different interferences. Data are presented as mean ± s.d. (*n* = 3). Significance was calculated by one-way analysis of variance. *P < 0.05.

Traditional self-BG monitoring does not have the problem of the glucose anomeric effect because the blood maintains high stability (pH 7.35–7.45). This is due to many protein and ion pairs acting as buffer ions in the blood. However, the pH of ISF fluctuates significantly, ranging from 6.6 to 7.6. When tissue is invaded by foreign matter, the inflammatory response is more likely to cause the local environmental pH to be as low as 6–6.4. This leads to severe glucose anomeric effects, resulting in inaccurate monitoring. The impact of adjusting the microenvironmental pH levels near the sensors was assessed as a function of the OCP measurements. By applying the formula, we established the relationship between the OCP and pH:
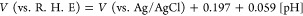


Because of the miniaturization and integration of both the working electrode and the reference/counter electrode within the microneedle structure, OCP decay transients serve as a reliable indicator of experimentally quantifying interfacial pH swings since the miniaturized distance between the working and counter electrodes, representing the potential difference concerning the reference electrode at equilibrium, thereby characterizing the pH of the local environment.^39^Figure 5a illustrates that the uncoated group demonstrated a linear correlation between the OCP and pH, with a slope close to the theoretical value of −0.059, indicating good follow-up to the formula. However, the OCP values of the Z-coated group were much more irrelevant to the bulk solution pH, with a flatter slope (−0.011). This is due to the self-prophetic-responsive nature of zwitterionic groups. In acidic environments, the zwitterionic polymer exhibits an overall cationic charge with NH_2_^+^ exposed, but SO_3_^–^ binds with environmental H^+^ (protonation) that is shielded internally. In alkaline environments, the polymer exhibits an overall anionic charge, with SO_3_^–^ exposed outward, and NH_2_^+^ loses H^+^ to form a neutral NH (deprotonation) that is then shielded inward. Therefore, under acidic conditions, zwitterionic groups consume protons from the environment, increasing the local pH; conversely, under alkaline conditions, they release protons, lowering the local pH. This mechanism works similarly to a buffer solution.^47−49^

To verify the study of glucose response by CGMs under different environmental pH values, we monitored uncoated and Z-coated CGMs at pH 7.6 and pH = 6.2. The results showed that the Z-coated CGM group exhibited an enhanced dynamic linear response to glucose levels, especially under low pH conditions (Figures 5b and S3). This robust support from Z-coating contributes to mitigating the anomeric effect of physiological glucose molecules in ISF, potentially ensuring the accuracy of CGM readings and reducing noise effectively, particularly during the acute inflammatory phase of implantation, where local pH may decrease to as low as 6.2–6.4.^42^

The general problem in the detection of glucose in ISF is the interference from physiological species, such as ascorbic acid (AA), uric acid (UA), and acetaminophen (AP). Introducing a negatively charged Nafion layer between the enzyme layer and conductive Pt electrode could prevent anion-type physiological species from interfering with the oxidation reactions. The selectivity for the uncoated and Z-coated CGMs against these possible interfering species was studied by the CA responses of successive physiological levels of various common electroactive species (0.11 mM AA, 0.4 mM UA, and 0.1 mM AP) and glucose (5 mM). The current responses to different physiologically electroactive molecules indicated that the Z-coated CGM showed an excellent anti-interference ability to all substances; however, the uncoated CGM displayed relatively poor anti-interference ability, especially for uric acid. This could be associated with the wealthy charged groups of zwitterionic coating^40^ (Figure 5d–f).

To simulate the environment of subcutaneous tissue, 3 wt % agarose was used as a skin prosthesis and soaked in glucose solutions of different concentrations. CGMs were inserted sequentially from low to high agarose, and the response current change was recorded. Z-coated exhibited significantly higher sensitivity than uncoated (Figure S4), presumably because of the compatible interface between the zwitterionic coating and agarose hydrogel. This allows glucose to move smoothly in and out of the layers, even in an environment with less solution. In addition, even after multiple plugging and unplugging processes, all of the CGMs maintained decent responses, indicating the robustness between the coated layers and electrode.

For in vivo rat experiments, the glucose levels of diabetic SD rats were monitored using uncoated and Z-coated CGMs. Notably, there existed a delay in the differences in glucose levels between the CGM systems and BG measurements. This delay results from glucose diffusion across the walls of capillary vessels and through the interstitial voids to the sensor. The average physiological time delay (BG to ISF glucose) is assumed to be 5–10 min. The delay can be observed during fluctuations in BG values, which impacts the accuracy of CGM readings. To address this issue, delayed compensation for BG readings to perform correlation dependence with CGM readings is necessary.^36^ In our experiments, the BG readings were artificially compensated by 300, 450, 600, and 750 s. The measured BG values were correlated with the CGM corresponding current points with 4 different delayed compensations to generate 4 sets of correlation plots and their corresponding correlation coefficients (Figure S5). The results showed that the correlation coefficients for the Z-coated CGMs were consistently higher than those for the uncoated CGMs, regardless of delay compensation. The highest correlation coefficient of 0.86 for the Z-coated sensor and 0.65 for the uncoated sensor was observed at a 450 s compensation, which was then used for subsequent data analysis.

**Figure 6:**
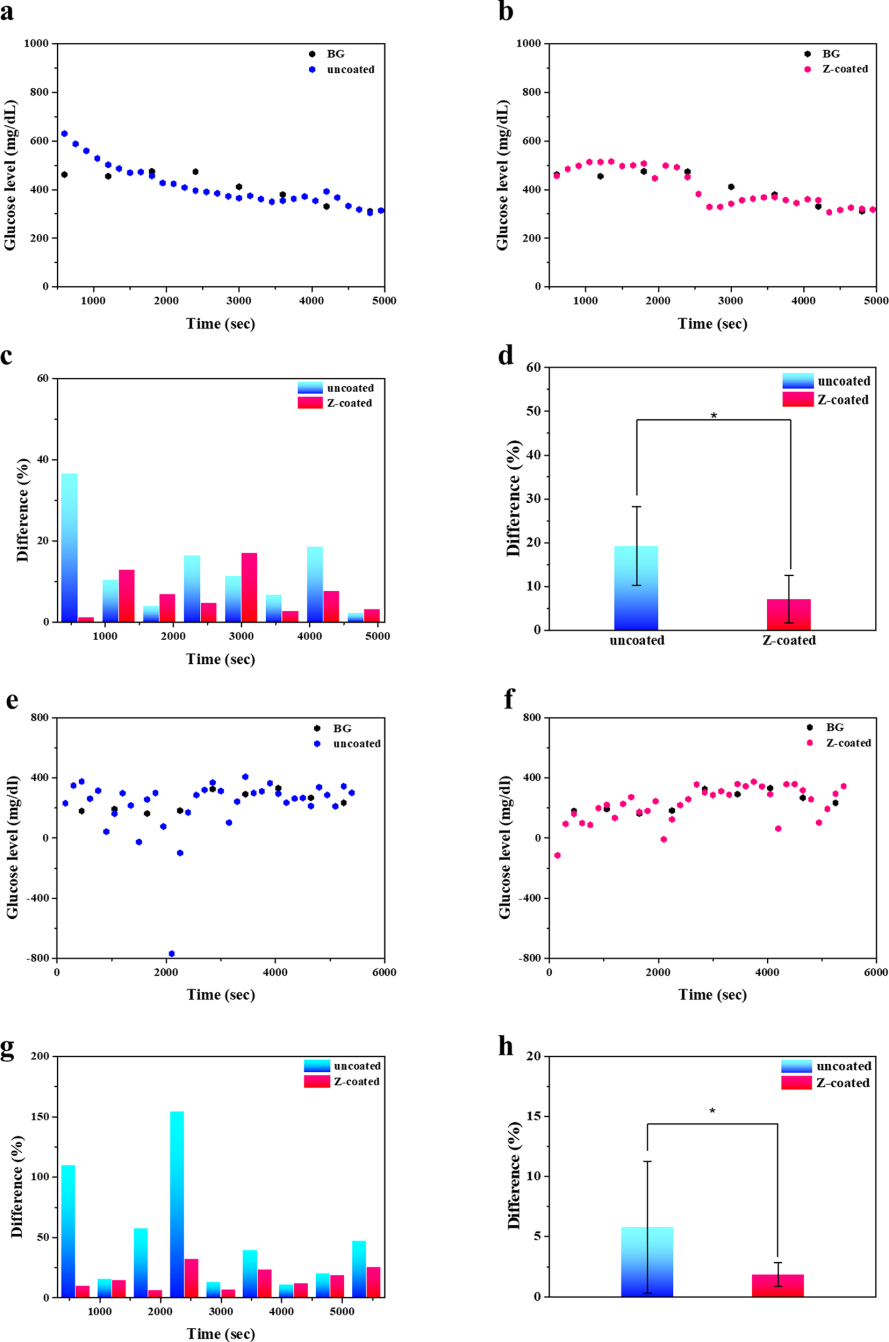
(a,b) Recalibrated glucose levels during the entire recording period in diabetic SD rats, using 450 s of compensation, compared with measured BG readings from Z-coated and uncoated sensors. (c) Deviation (% difference) between recalibrated glucose levels and measured BG for Z-coated and uncoated sensors in diabetic SD rats. (d) Summary of significance for percentage differences in uncoated and Z-coated CGMs in diabetic SD rats. (e,f) Recalibrated glucose levels during the entire recording period in normal SD rats, using 450 s of compensation, compared with measured BG readings from Z-coated and uncoated sensors. (g) Deviation (% difference) between recalibrated glucose levels and measured BG for Z-coated and uncoated sensors in normal SD rats. (h) Summary of significance for percentage differences in uncoated and Z-coated CGMs in normal SD rats. Data are presented as mean ± standard deviation (*n* = 9). Significance was calculated using one-way ANOVA. *P < 0.05.

The generated linear regression equations from the correlation plots can be applied to fit sensor signal values to BG values, allowing glucose levels to be obtained based on the recorded electrical signal at the corresponding times.^36^ The glucose level versus BG over time was then replotted (Figure 6a,b). These results indicated that the recalibrated concentration points to BG readings for the Z-coated CGM showed a better and tighter follow-up than the uncoated CGM. For all sensors, the recalibrated glucose trends were compared with measured BG values at the appropriate corresponding time points, and their deviation (% difference) from BGs is shown in Figure 6c. The Z-coated CGM displayed a significant reduction of noises in % difference compared to the uncoated CGM at all corresponding times. Comparisons of recalibrated glucose levels versus measured BG values during the recording period are statistically summed, as shown in Figure 6d. The uncoated CGM showed 19.25 ± 7.03% inaccuracy of recalibrated glucose levels compared to BG readings. However, the inaccuracy significantly decreased to 8.99 ± 5.42% for the Z-coated CGM.

For healthy SD rats, the highest correlation coefficient of 0.83 for the Z-coated sensor and 0.54 for the uncoated sensor was observed at a 450 s compensation (Figure 6e,f). The uncoated CGM showed 52.13 ± 49.43% inaccuracy of recalibrated glucose levels compared with BG readings, and the inaccuracy significantly decreased to 16.64 ± 8.98% for the Z-coated CGM (Figure 6g,h). It was strongly evident that after recalibration, the Z-coated sensor outperformed the uncoated sensor in both trend accuracy and the capability of noise reduction for both diabetic and healthy SD rats, suggesting that the zwitterionic coating significantly improves the accuracy of CGMs, associated with the combined effect of adjusting the microenvironmental pH and the antifouling ability at the acute inflammatory phase. Furthermore, even for the non-recalibrated data (Figures S6–S9), the Z-coated CGM exhibited a significant reduction in noise and a good follow-up (by using a polynomial fitting) with a higher correlation coefficient to BG readings than the uncoated CGM at all delayed compensations for both diabetic and healthy rats.

Lastly, histological examinations were conducted on tissues surrounding implanted CGMs to investigate the anti-inflammatory nature of Z-coating. Tissues surrounding uncoated and Z-coated CGMs implanted in SD rats for 1 day were infiltrated and stained using hematoxylin and eosin (H&E) and Masson’s Trichrome staining. In the H&E-stained sections, tissues adjacent to the uncoated CGM exhibited significantly darker staining than other regions, probably due to the accumulation of proteins, neutrophils, and mast cells.^43^ In contrast, the overall tissue response was less severe around the Z-coated CGM. Masson’s Trichrome staining revealed that the degree of fibrosis, indicated by the muscle fibers (red) and collagen fibers (blue), was more pronounced in the tissues surrounding the uncoated CGM compared to the Z-coated CGM. Fibrosis typically occurs in later stages of inflammation, suggesting that the initial tissue injury from CGM implantation caused the observed muscle and collagen fiber damage (Figure S10). Stained section images demonstrated that the zwitterionic coating significantly reduced the tissue response following CGM implantation, even during the most intense early inflammatory phases. This is because the super hydrophilicity of the zwitterionic coating reduces surface protein adsorption, effectively minimizing the inflammatory response.

Due to noise in the implantable monitoring process, CGM users must regularly perform fingertip blood sampling to compensate for errors caused by noise and artifact drifting. This practice causes patient discomfort and hinders CGM independence from traditional BG self-monitoring. Noise sources include tissue changes (inflammation or edema), fluctuating pH environments, sensor lifespan (fibrosis process and electrode passivation), and unstable diffusion, all of which can cause CGM readings to deviate from BG levels. In this study, the zwitterionic brush-like coating has been shown to reduce postimplantation inflammation and regulate the pH of the implant localized microenvironment, which is highly beneficial for obtaining accurate glucose monitoring during initial implantation inflammation and fluctuating pH in ISF. Additionally, the zwitterionic coating slows the hydrolysis of the sensor and stabilizes interface diffusion.

Overall, the experimental results suggest that modifying the zwitterionic layer on CGMs using atmospheric plasma technology could address sensor noise, potentially eliminating the dependence on daily BG repetitive finger prick calibration. However, there are still some limitations to using plasma technology. For example, it is difficult to control the thickness of the zwitterionic layer to optimize sensor performance. Second, appropriate parameters require further testing, as excessive power can deactivate enzymes, while insufficient power can lead to poor grafting with an unreliable product yield. Third, due to plasma technology’s limitations, uniform coating coverage on nonplanar electrodes may not be achievable. Future improvements may include (1) developing ultra-adhesive materials (such as polydopamine) as intermediate adhesive layers to connect the glucose limiting layer and the zwitterionic coating and (2) using inert gas-based mild plasma to regulate plasma parameters to optimize sensor performances.

## Conclusions

In summary, we have successfully demonstrated that the zwitterionic brush coating via atmospheric plasma-induced grafting can not only adjust the localized microenvironmental pH to mitigate the glucose anomeric effect and ensure monitoring accuracy but also extend the lifetime of CGM due to the protection from bulk water erosion on the glucose confinement layer. The Z-coated CGM displayed a better follow-up to BG levels with higher correlations and a significant reduction in noises (difference to BG levels), regardless of non-recalibration and after recalibration. These results suggested that the zwitterionic brush coating significantly improves the performance and accuracy of CGMs, especially at the stage of acute inflammation when initially implanted.

This work was financially supported by the Ministry of Science and Technology of the Republic of China, Taiwan, under Contract No. MOST 111-2222-E-A49-006 -MY2. The authors thank HMD Biomedical Incorporation for providing standard blood glucose meter kits (GlucoLeader GV01) and the Taiwan Animal Care and Use Committee and National Yang-Ming Chiao Tung University for animal use and care approval.

## Supporting Information Available

In vivo animal setup; XPS full spectrum; chronoamperometry responses among different pH environments; glucose responses in agarose; correlation between CGM to BG signals at different delayed compensations; deviation in difference at different delayed compensations; and tissue images of H&E staining and Masson’s trichrome staining (PDF)
