# Supplementary material for: Tuning the Localized Microenvironment near a Continuous Glucose Meter to Ensure Monitoring Accuracy and Longevity by Plasma-Induced Grafting Zwitterionic Brushes
Source: ACS Sens. 2024 Dec 5;9(12):6520–30. doi: 10.1021/acssensors.4c01921 (PMC11686511; doi:10.1021/acssensors.4c01921)
Supplement: Supplementary file 1 — se4c01921_si_001.pdf [file se4c01921_si_001.pdf]

Tuning the Localized Microenvironment Near a Continuous Glucose Meter to Ensure Monitoring Accuracy and Longevity by Plasma-Induced Grafting Zwitterionic Brushes

Syuan-Jia Shin[1], Pei-Chen Lo[1], Yen-Ting Wu[1], Huai-Hsaun Shao[1], Dai-Jin Li[1], Yung-Cheng Weng[1], You-Yin Chen[1], Ta-Chung Liu[1,*]

[1]Department of Biomedical Engineering, National Yang Ming Chiao Tung University, 155 Lin-Ong St., Taipei, Taiwan 11221, ROC.

[*]Correspondence should be addressed to either of the following:

Prof. Ta-Chung Liu

Department of Biomedical Engineering, National Yang Ming Chiao Tung University, 155 Lin-Ong St., Taipei, Taiwan 11221, ROC.

Tel: + 886-2-2826-7000 67019

Email: tcliu@nycu.edu.tw

Supporting Information

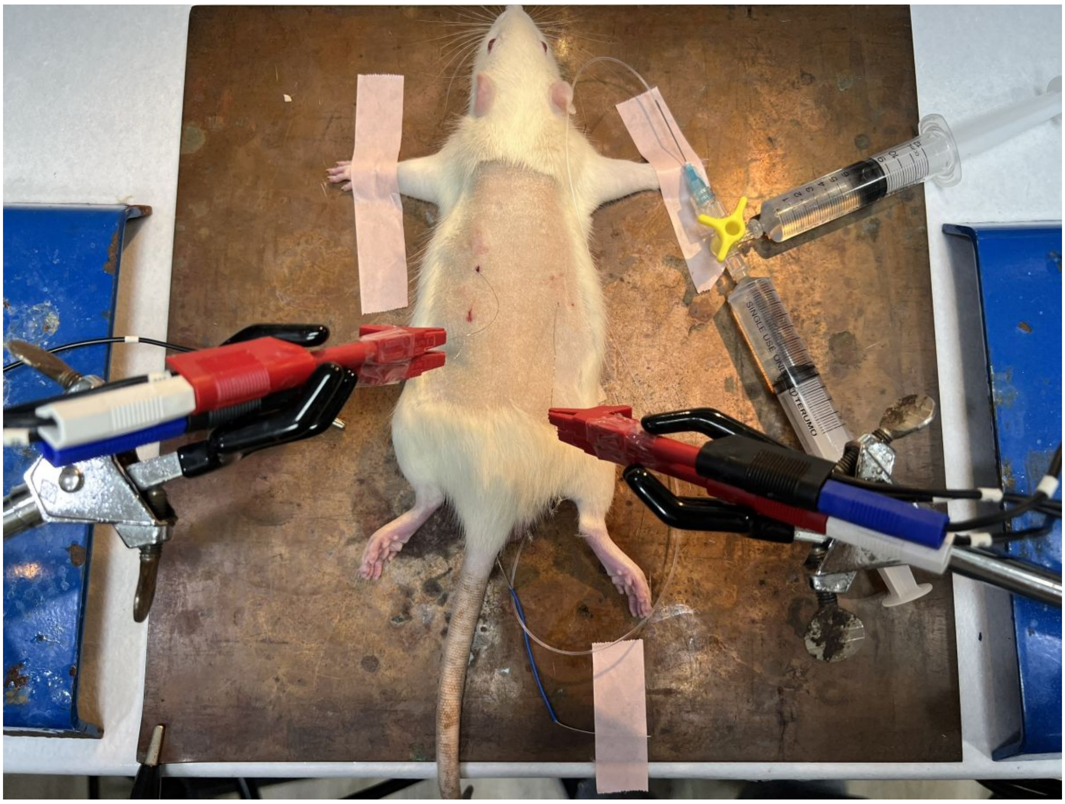

Figure S1. *In vivo* animal setup of Z-coated and uncoated sensors.

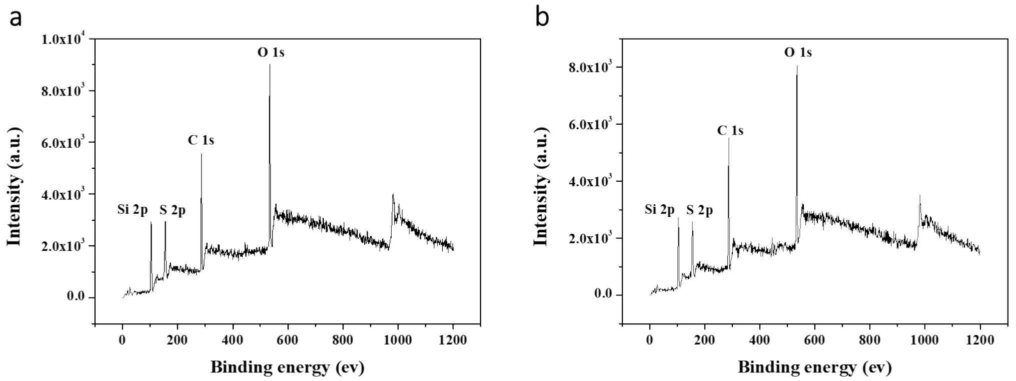

Figure S2. XPS full spectrum for (a) uncoated and (b) Z-coated sensors.

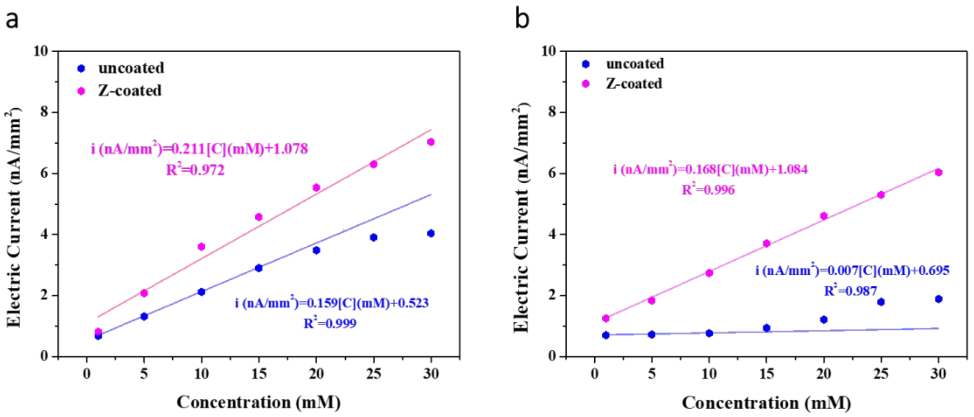

Figure S3. CA responses (at 0.6 V vs. Ag/AgCl) for the uncoated and the Z-coated CGM by sequentially injecting from glucose-free to 20 mM at pH of (a) 7.6, and (b) 6.2, respectively.

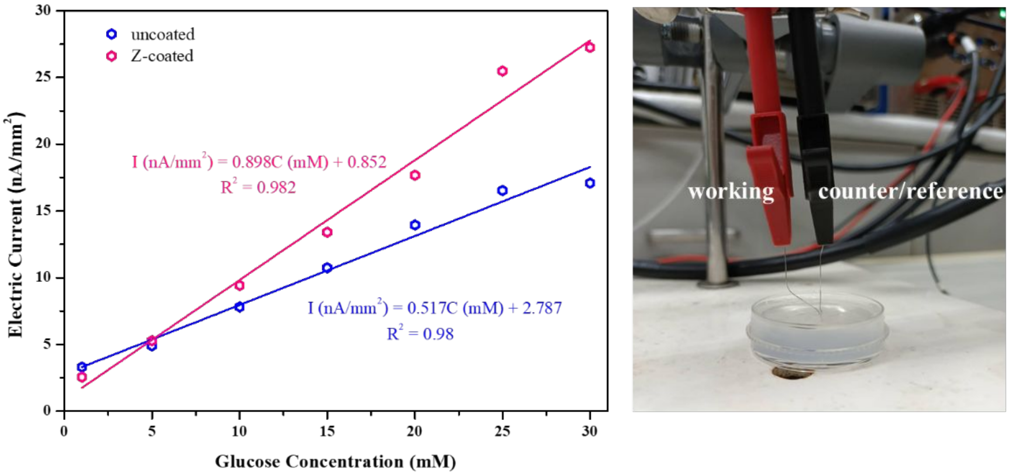

Figure S4. Performance of CGMs in agarose with different glucose concentrations.

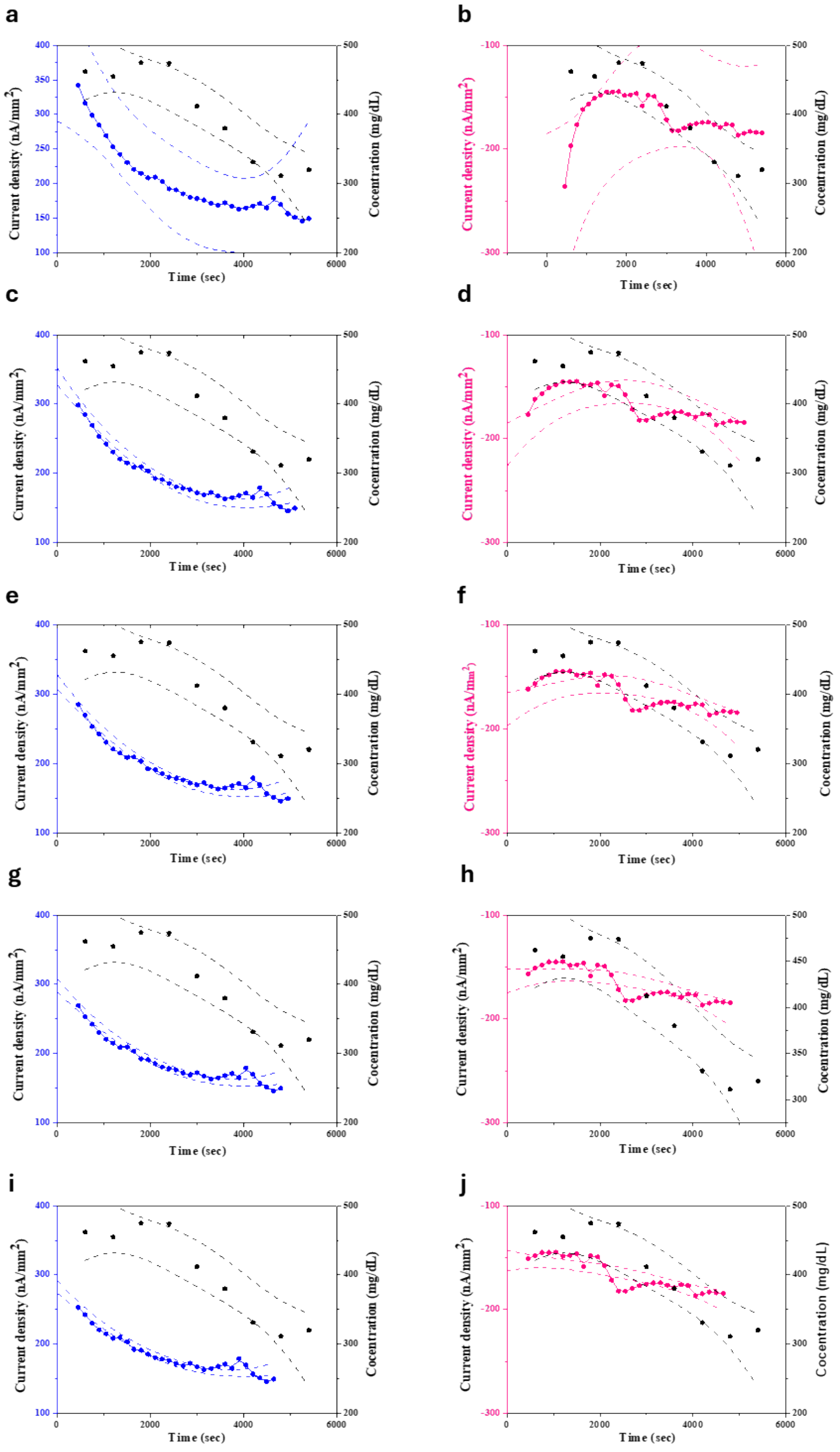

Figure S5. Comparisons of correlation of the CGM signal to BG levels at different delayed compensation by 0, 300, 450, 600, and 750 seconds for uncoated CGM (a, c, e, g, i) and Z-coated CGM of diabetic SD rat (b, d, f, h, j).

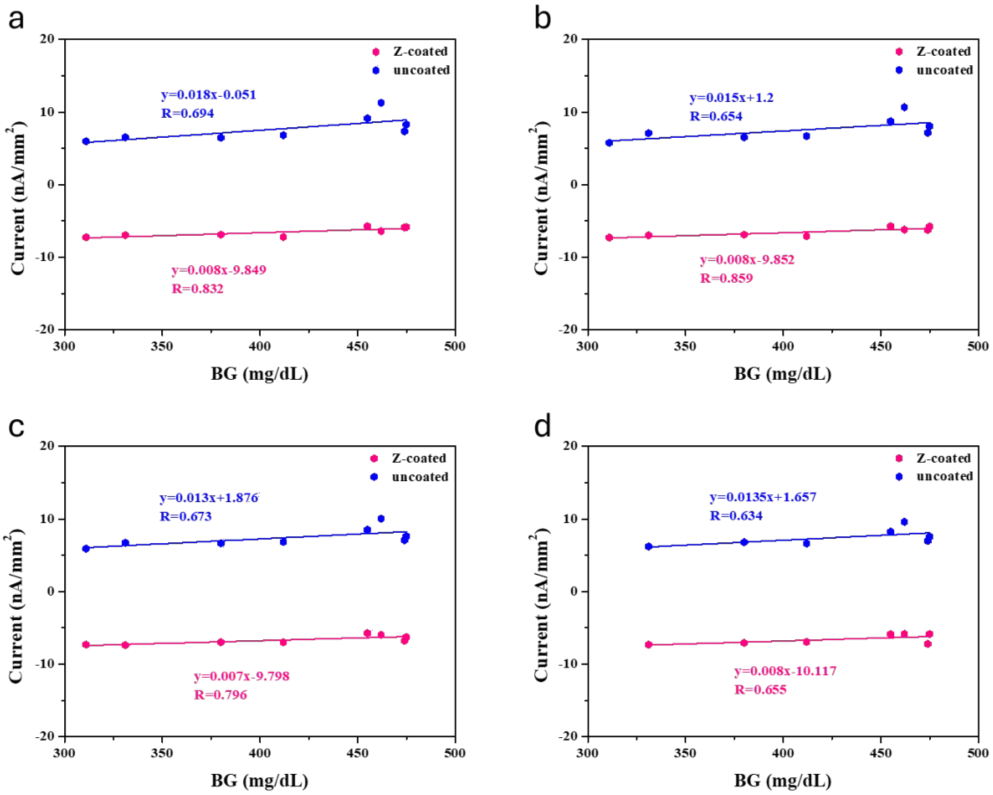

Figure S6. Correlation and their linear regression plots of uncoated and Z-coated CGMs with delayed compensation of (a) 300, (b) 450, (c) 600, and (d) 750 seconds of diabetic SD rat.

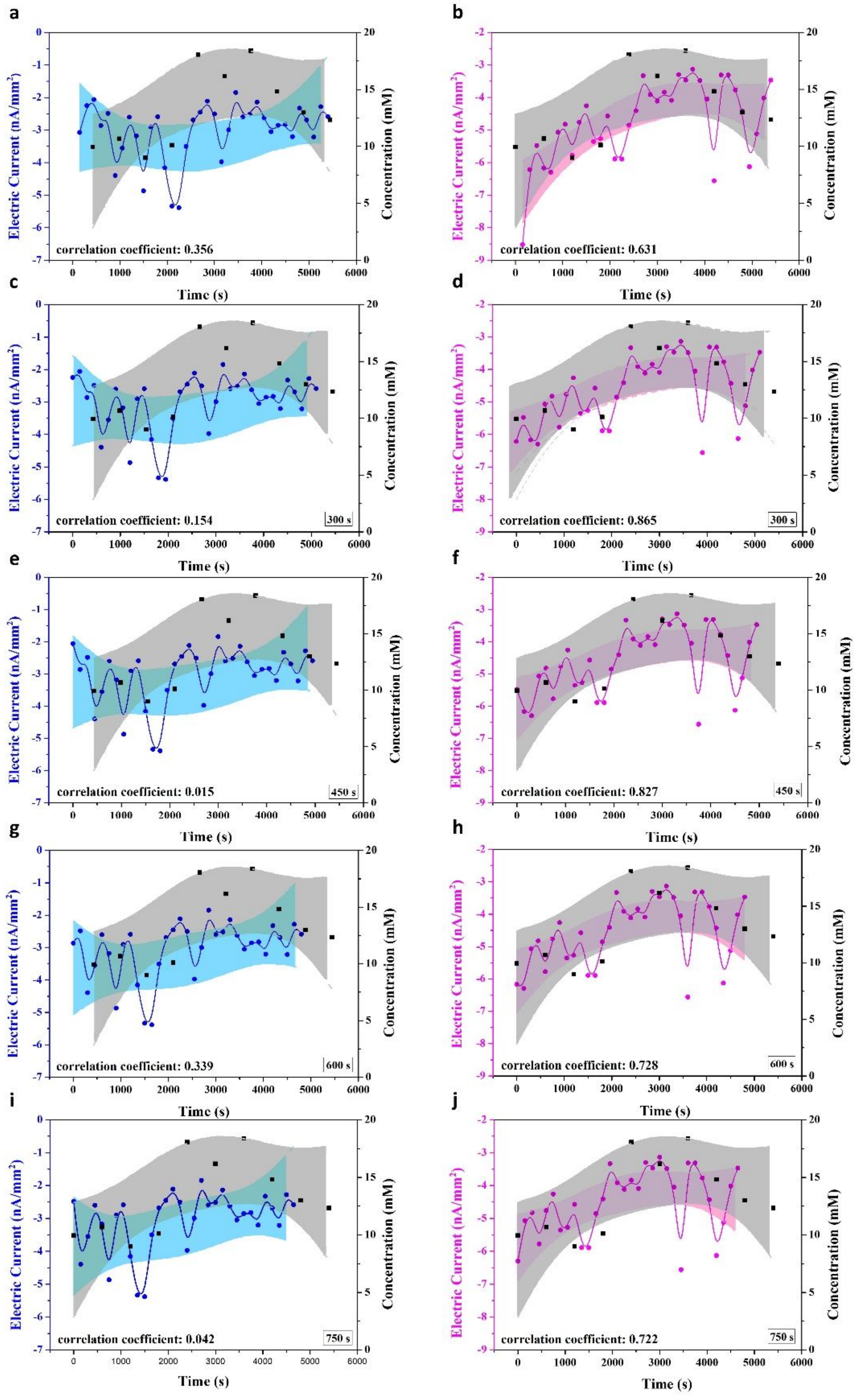

Figure S7. Comparisons of correlation of the CGM signal to BG levels at different delayed compensation by 300, 450, 600, and 750 seconds for uncoated CGM (a, c, e, g, i) and Z-coated CGM of healthy SD rat (b, d, f, h, j).

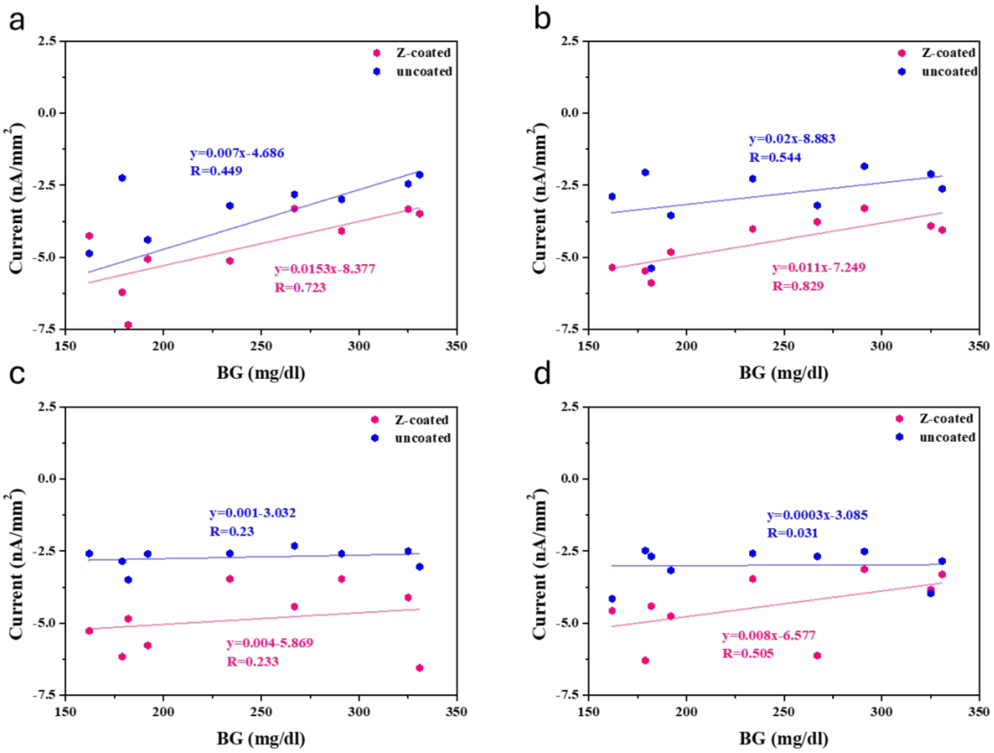

Figure S8. Correlation and their linear regression plots of uncoated and Z-coated CGMs with delayed compensation of (a) 300, (b) 450, (c) 600, and (d) 750 seconds of healthy SD rat.

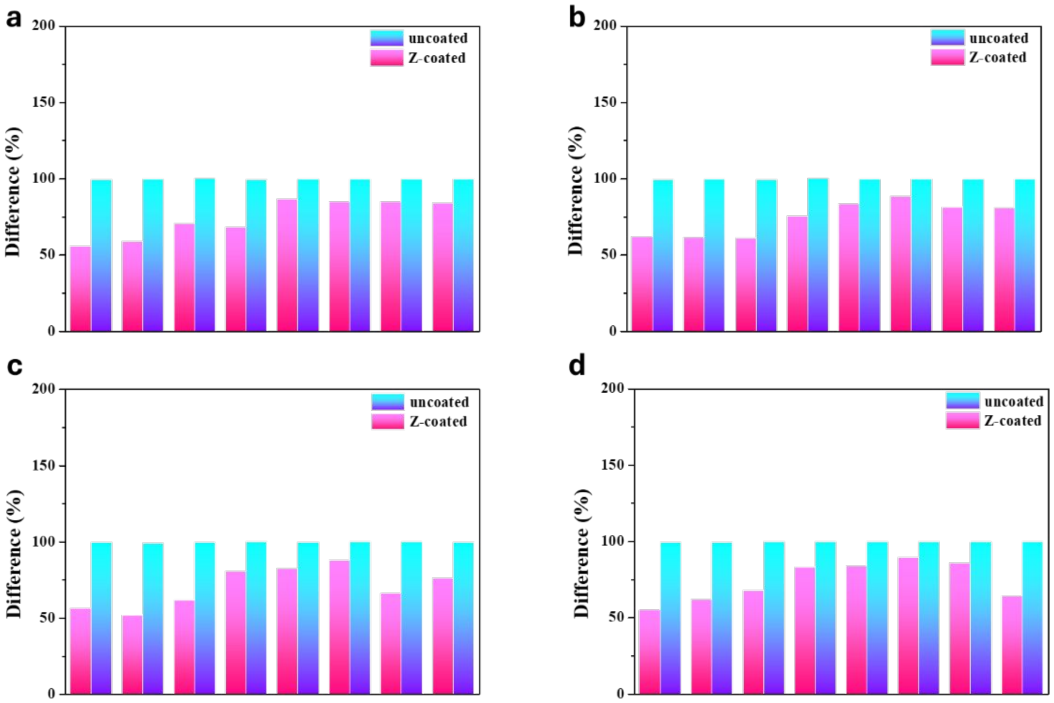

Figure S9. The deviation in percentage difference plots of uncoated and Z-coated CGMs with delays of (a) 300, (b) 400, (c) 600, and (d) 750 seconds without recalibration for heathy SD rat.

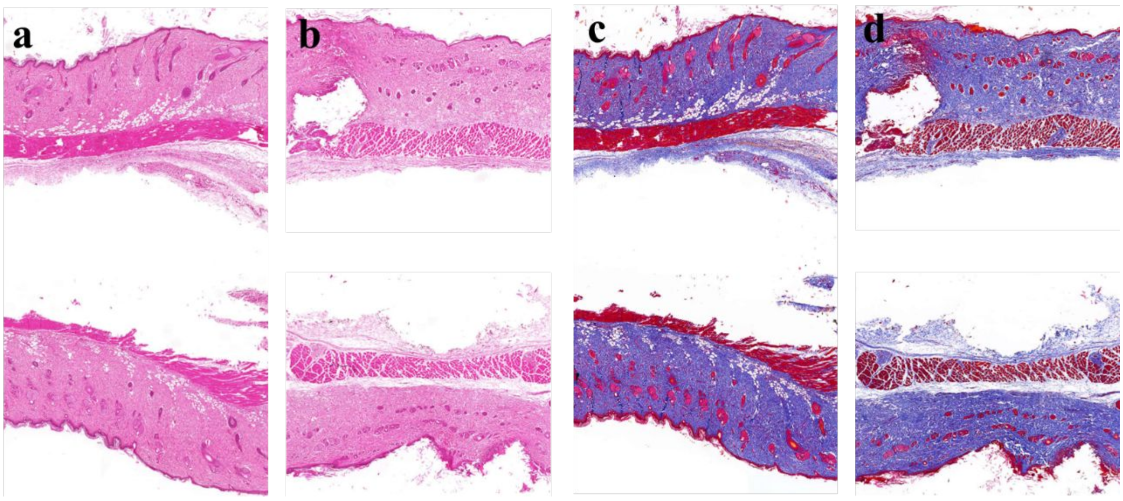

Figure 10. (a-b) Tissue images of H&E staining of uncoated CGM and Z-coated CGM. (c-d) Masson's trichrome staining tissue images of uncoated CGM and Z-coated CGM (400X).

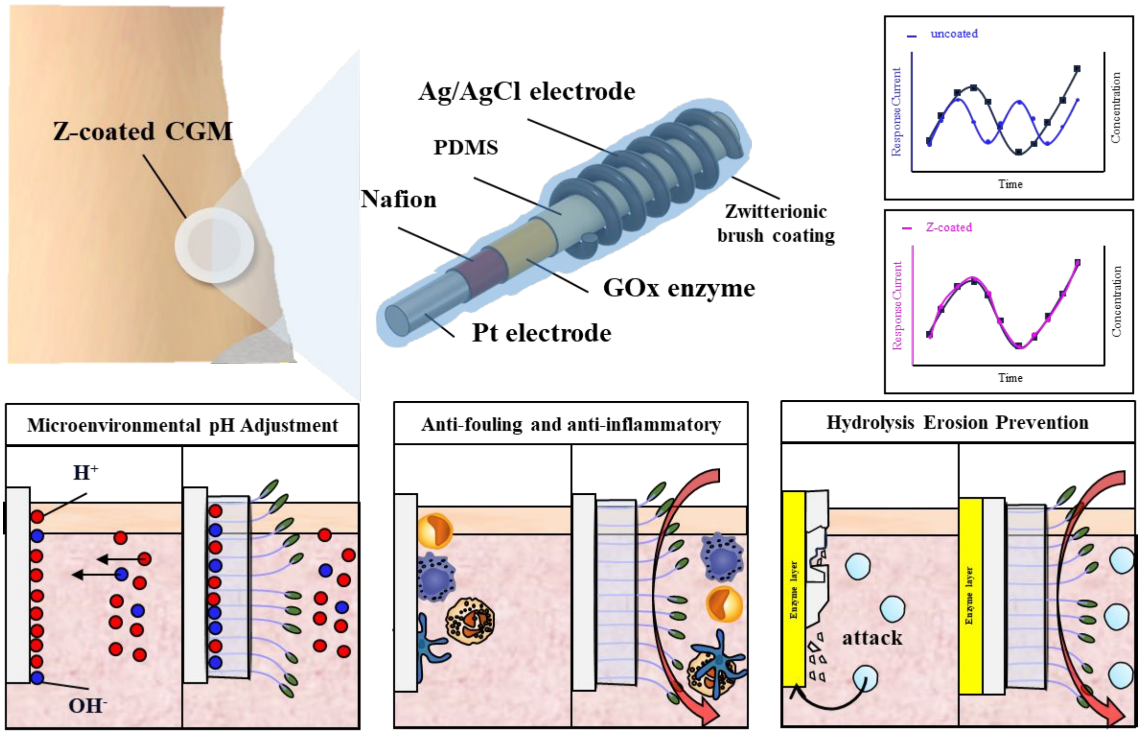

For Table of Contents Only.
